# Using Sensors and Unmanned Aircraft Systems for High-Throughput Phenotyping of Biomass in Perennial Ryegrass Breeding Trials

**DOI:** 10.3389/fpls.2019.01381

**Published:** 2019-10-30

**Authors:** Junping Wang, Pieter Badenhorst, Andrew Phelan, Luke Pembleton, Fan Shi, Noel Cogan, German Spangenberg, Kevin Smith

**Affiliations:** ^1^Agriculture Victoria Research, Hamilton, VIC, Australia; ^2^Agriculture Victoria Research, AgriBio, Bundoora, VIC, Australia; ^3^School of Applied Systems Biology, La Trobe University, Bundoora, VIC, Australia; ^4^The Faculty of Veterinary and Agricultural Sciences, The University of Melbourne, VIC, Melbourne, Australia

**Keywords:** *Lolium perenne*, normalized difference vegetation index, perennial ryegrass, sensor, unmanned aerial vehicle, biomass

## Abstract

Increasing herbage biomass is the predominant objective for pasture plant breeding programs. Three types of field trials are commonly involved during forage plant breeding, i.e., individually spaced plants, row plot, and sward trials. Assessments of biomass production at individual plant, row plot, and sward plot levels are through visual scoring and/or cutting of biomass manually or mechanically. Both visual scoring and cutting of plants are laborious, time consuming, and costly. The development of sensor technology such as multispectral sensors and unmanned aircraft systems (UAS) provide the opportunity to accelerate the process of biomass evaluation and to increase throughput, improve resolution, and reduce time and cost. We tested either the handheld Trimble GreenSeeker^®^ or Parrot Sequoia multispectral sensors attached to a 3DR Solo Quadcopter to assess biomass in perennial ryegrass field trials sown as spaced individual plants, row plots, and simulated sward plots. Significant correlations were observed between visual score and normalized difference vegetation index (NDVI) in a spaced plant field trial and between biomass yield and NDVI in row plot and sward trials (r = 0.12 ~ 0.93). NDVI obtained from multispectral sensors and UAS can replace visual scoring in spaced plant trials. It was also a valuable proxy for yield estimation in row plot and sward trials. These technologies will assist in transition for the forage grass breeding from pen and notepad to digital and data era.

## Introduction

Increasing herbage biomass production is the predominant objective for pasture plant breeding programs. The common breeding systems include ecotype selection, restricted recurrent phenotype selection, half-sib progeny test, between-and-within family selection, and recurrent multistep family selection (Vogel and Pedersen, 1993). Whichever breeding systems are adopted, there are three types of field trials generally involved: transplanted spaced plant nursery, which allows breeders to observe variation and make selection within a population/family/accession; clonal row trials, which test the performance of polycross clonal progenies; and seeded sward plot trials, which enable the evaluation of population/family/cultivar performances (Humphreys et al., 2010; Hayes et al., 2013). The goal of breeding trials is to select the best genotypes or lines within a group of selection candidates. Therefore, the ranking order based on biomass yield of the candidates compared to reference cultivars and each other is often the primary focus rather than the absolute yield.

The method for evaluation of spaced plant nursery trials and row trials is commonly based on breeder’s visual score, which gives a discrete rank order of 1 to 5 or 1 to 9. Although, the visual score is much more efficient than cutting plants, drying, and weighing the biomass (Smith et al., 2001), it is highly subjective and is influenced by interference from surrounding plants. The method for evaluating biomass of sward trials generally involves sampling and cutting plants either manually or mechanically. In each breeding cycle, repeated evaluations over seasons and years are essential for perennial grasses. The cost in time and labor becomes a restricting factor for the upscaling of any breeding program. The inability to accurately screen large numbers of plants is one of the limitations to increase the rate of genetic gain in forage grasses.

Technologies for rapid, nondestructive, and high-throughput biomass evaluation have been highly sought after especially when next-generation genotyping and sequencing becomes available and requires the in-field phenotyping to be high throughput to support genomic selection programs. Remote sensing is often used to assess rangeland condition and primary productivity across large areas on earth surface using satellite platforms (Pettorelli et al., 2005). Normalized difference vegetation index (NDVI) provided a proxy measure for green plant biomass (Tucker et al., 1981; Schino et al., 2003). Adaptation of NDVI into field-based spectrometry makes the application into breeding scale trials possible. The ground-based Trimble GreenSeeker^®^ (Trimble Navigation Limited, Sunnyvale, CA, USA) optical sensor emits light at two fixed wavelengths (660 ± 10 nm and 770 ± 15 nm) and measures the amount of each type of light that is reflected from the plant and outputs the calculated NDVI value. Using NDVI obtained by GreenSeeker as a biomass proxy has been reported for a range of crops (Teal et al., 2006; Li et al., 2010; Raun et al., 2011; Lofton et al., 2012; Ji et al., 2017). The Trimble GreenSeeker can be either handheld or mounted on a ground-based vehicle such as buggies or tractors to increase the throughput (Deery et al., 2014).

The development and adoption of unmanned aircraft systems (UAS) provide an airborne platform for high-throughput phenotyping, which can be at centimeter-level resolution (Shi et al., 2016). Attached with various sensors, it has been used for high-throughput in-field phenotyping of plant biomass accumulation (Busemeyer et al., 2013) and responses to drought (Ludovisi et al., 2017). The aerial-based NDVI and ground-based NDVI are highly correlated (Duan et al., 2017). The selection of either ground or airborne platforms will depend on the trial scale. Aerial-based platforms are more suitable for large scale trials in which postimage processing is essential and critical. Ground-based platforms are suitable for smaller trials, where walking/driving through the trial is logistically possible within a few hours and do not require postprocessing of images. As pointed out by Shi et al. (2016), most research to date has been on “one-off” projects to demonstrate the technology and stopped short of developing routine methods. The applicability of these sensors and platforms for forage grass breeding and how they can be applied routinely remains to be validated. Since 2014, we have developed ground- and aerial-based platforms for nondestructive high-throughput forage grass phenotyping, firstly for biomass yield, here, we validate these methods on our prebreeding research trials to facilitate genomic prediction and selection in ryegrass.

In this paper, we describe using Trimble GreenSeeker and multispectral sensors attached to UAS for high-throughput in-field biomass phenotyping in our perennial ryegrass pre-breeding research trials. These trials represent all three trial types of spaced plant, row plot, and seeded sward trials in forage grass breeding. The goal is to validate the ground-based and aerial-based platforms for nondestructive high-throughput biomass phenotyping and their potential to replace traditional visual score and clipping for routine application in breeding to improve data collection and decision-making ability.

## Materials and Methods

### Field Trials

For this study, we used perennial ryegrass as the model species for perennial forage grasses. Three types of breeding trials were used to validate the sensors and platforms. All field trials were conducted in the research farm in Hamilton, Victoria, Australia (–37.841S, 142.073E). A spaced plant trial of perennial ryegrass was used to test the correlation between NDVI and visual score and therefore the possibility to replace visual scoring for plant biomass/vigor in a spaced plant nursery. This trial contained a total of 2,576 individual plants in four blocks. Each block contained 644 individual genotypes randomly assigned in a 46-row by 14-column layout. The spacing was 60 cm between columns and 40 cm between rows. The primary aim of this trial was to screen genotypes for drought tolerance. Therefore, two blocks received natural rainfall as control and two blocks were under rainout shelters, which received less rainfall as a drought treatment. Here, the trial was used to develop the relationship between the aerial-based NDVI and visual score and compare the selections based on the two measurements. The effect of drought stress and response of different genotypes to the stress will be reported in a subsequent manuscript. The trial was planted in October 2015.

A row plot trial consisting of 50 perennial ryegrass cultivars/breeding lines with 10 replicates, a total of 500 plots, was used to test the correlation between biomass yield and aerial-based NDVI at plot level. Each plot consists of 96 plants from one cultivar, arranged in three rows and 32 plants per row. Distance between plot and rows within plot was 60 cm and distance between plants within a row was 25 cm. The area of the trial site was 8,100 sqm. The trial was transplanted in June 2016.

Two simulated sward trials of perennial ryegrass were used to test the correlation between NDVI obtained by handheld Trimble GreenSeeker with biomass yield. The first sward trial was a cultivar subselection trial which contained 60 plots. They were divided into 10 replicates each consisted of four subpopulations and two plots of the original cultivar. Each plot was a minisward that comprised 100 plants in a 10 plant × 10 plant, square grid layout. The distance between plants was 15 cm, which simulates the common spacing between rows as seeded sward. The distance between plots is 1 m. The trial was planted in May 2014. The second simulated sward trial was a perennial ryegrass F_2_ family trial, which contained 72 plots of 10 families of two generations and three reference cultivars in two replicates. The distances between and within plots were the same as the cultivar subselection trial. This trial was planted in May 2015.

### Data Collection

For the spaced plant trial, the individual plant from each block were visually scored on a scale from 0 to 9; where 0 (dead plant) and then 1 (the lowest biomass yield) to 9 (the highest biomass yield) on 12 December 2015 (before stress) and 28 July 2016 (after stress). The flight missions were conducted with a Parrot Sequoia multispectral sensor attached to 3DR Solo Quadcopter at a flight height of 20 m (with ground sampling distance of 2 cm, overlap and side lap 80%) on the same day as the visual score were taken. The Parrot Sequoia multispectral sensor has green (550 nm), red (660 nm), red edge (735 nm), and near infrared (790 nm) lenses all with a bandwidth of 40 nm along with a standard RGB camera, GPS sensor and incident light sensor. Images captured during takeoff and landing were discarded from further processing. Images processing, georectification and radiometric calibration were conducted through Pix4Dmapper (Version 4.1.15, Pix4D SA, Lausanne, Switzerland). Individual plant identification was achieved through an in-house developed segmentation algorithm in an R environment. NDVI for each plant was extracted from the reflectance at red and near infrared wavelengths represented in a reconstructed and segmented orthomosaic through QGIS.

**Figure 1:**
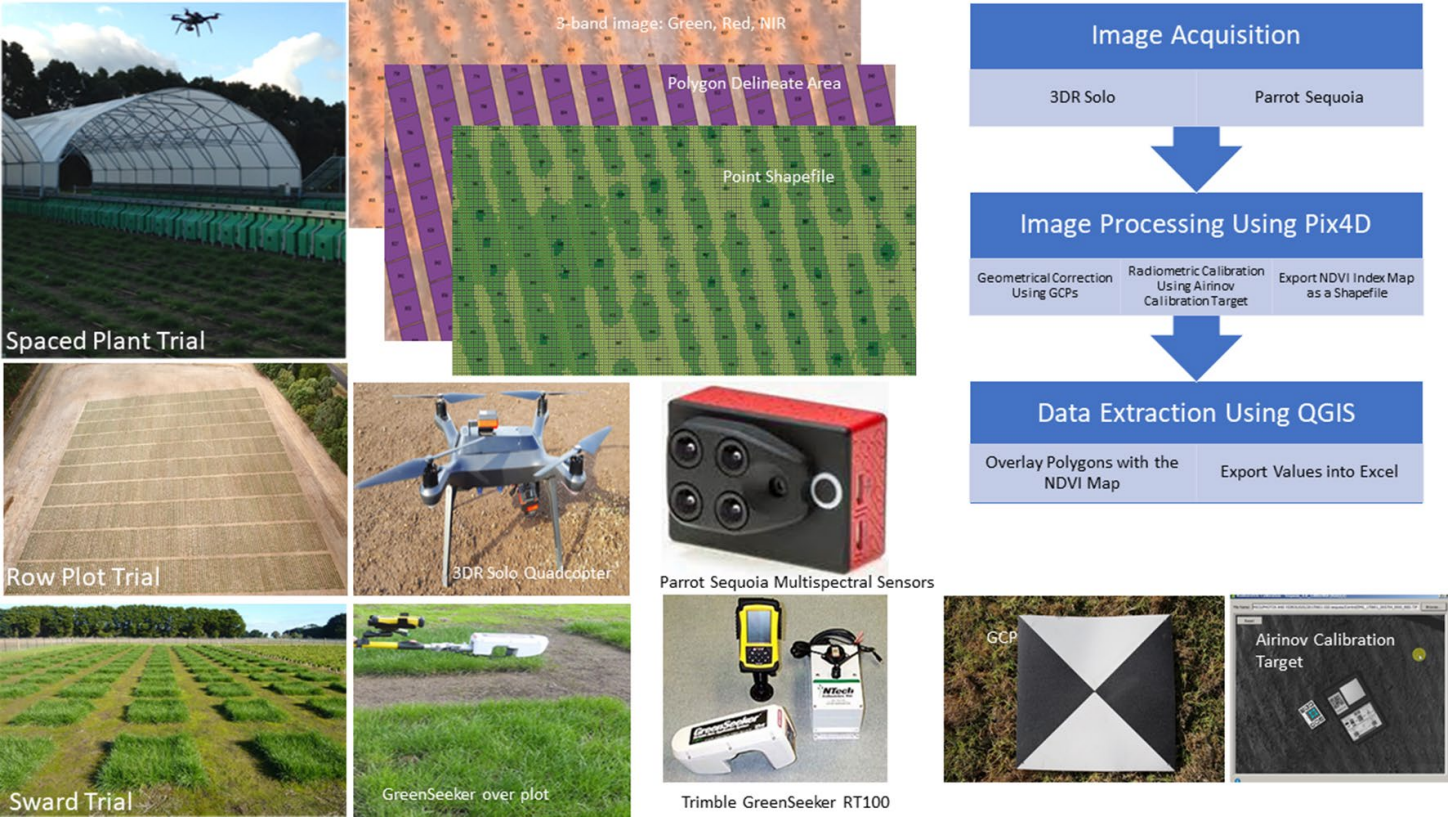
Three types of field trials and schema of data collection. The normalized difference vegetation index (NDVI) was extracted from the spaced plant trial and row plot trial by aerial imaging using 3DR Solo Quadcopter and Parrot Sequoia multispectral sensors and image analysis. The NDVI values from the sward trials were measured using Trimble Greenseeker RT100.

For the row plot trial, a weekly flight missions were undertaken with the 3DR Solo multirotor and Parrot Sequoia multispectral sensor at the same flight height, speed, and overlap as described for the spaced plant nursery above. Image process followed the same procedure as described above (**Figure 1**). Segmentation was conducted for each row and NDVI was extracted at the row level. NDVI of each plot was averaged of the three rows. Mechanical harvests were conducted at row plot level and biomass yield in fresh weight was recorded to test the correlations between biomass and NDVI on four occasions.

For the simulated sward trials, herbage samples were harvested when plants reached 2.5- to 3-leaf stage with a push mower at a height of 5 cm from the ground. Biomass yield in fresh weight for each plot was recorded, and for most of the harvests, 200–300 g of herbage was subsampled from each plot and the subsamples oven-dried at 60°C for 48 h and dry matter yield of every plot was calculated. A total of 16 harvests were conducted for the cultivar subselection trial and 15 for the perennial ryegrass F_2_ trial. NDVI values were collected weekly by walking through the trial plots with a handheld Trimble GreenSeeker RT100 system at 80 cm height over the plot. The NDVI value of each plot was averaged from approximately 20–30 readings of the plot.

### Statistical Analysis

Statistical analysis was conducted using GenStat (Payne et al., 2009). Pearson correlation coefficients between NDVI and reference data were obtained using correlation command in GenStat for all field trials in each harvest. For the spaced plant trial, the best linear unbiased prediction (BLUP) of genotypic effects and tests of significance were conducted using residual maximum likelihood (REML) model in GenStat with genotype and replicate were fitted as random effects under both control and drought conditions. To compare the selection based on visual score and NDVI for the spaced plant trial, we assumed two scenarios. In the first scenario, the top 50 genotypes would be selected from each block (treat each block as independent) based on the raw data. In the second scenario, the 50 highest score/NDVI genotypes would be selected under both control and treatment conditions based on the BLUP values. The maximum consistency was calculated as the most possible number of common genotypes selected based on the two measurements. For the row plot trial, REML was used to analyze the data as a linear mixed model with cultivar fitted as a fixed effect and experimental design factors (column and row) as random effects. The cultivar ranks based on the predicted mean of yield and NDVI at each harvest were compared.

## Results

### Relationship Between NDVI and Visual Score From the Spaced Plant Trial

**Figure 2:**
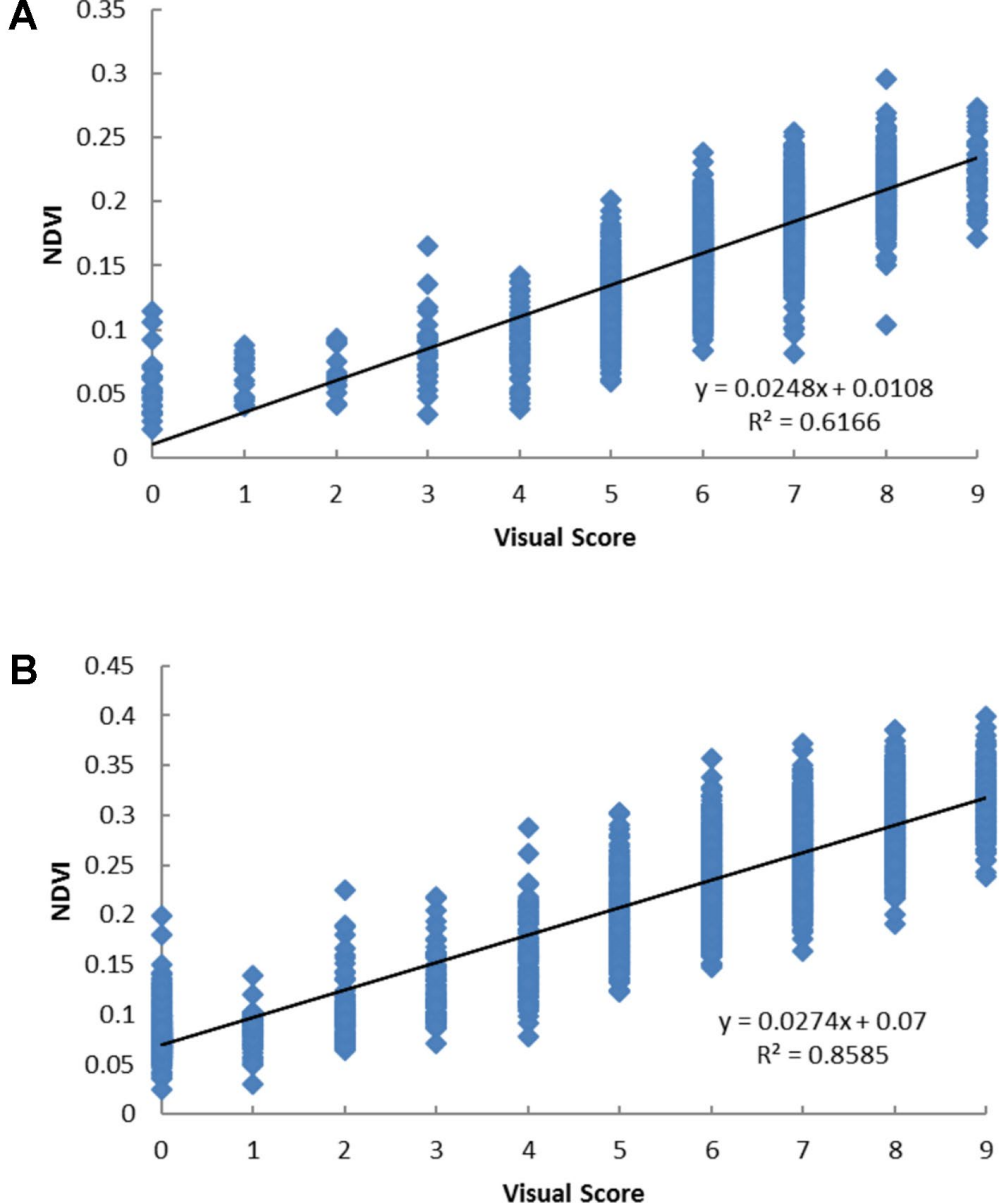
The scatter plots of visual score and the normalized difference vegetation index (NDVI) values based on Parrot Sequoia multispectral sensor attached to 3DR Solo Quadcopter in perennial ryegrass spaced plant trial in December 2015 **(A)** and July 2016 **(B)**.

Significant correlations (p < 0.001) were observed between NDVI and visual score and the correlation coefficients were 0.79 and 0.93 in December 2015 and July 2016, respectively (**Figures 2A, B**). Each visual score spanned a range of NDVI values and there were considerable overlaps of the range of NDVI values across different visual score groups.

In the first scenario of selection, 50 genotypes were to be selected from each block to approximate a selection strategy that a breeder may use. Based on the distribution of the visual scores of the four blocks (**Table 1**), if selection was made for the top 50 plants from each block based on visual score, plants in the same ranking may be chosen randomly. For example, based on vigour on July 2016, for field block-1 the 24 plants ranked “9” would be selected and the remaining 26 genotypes would be selected from the next level of rank “8” which had 115 plants (**Table 2**). If selection was made based on NDVI values, the genotype selection would be certain due to NDVI being a continuous variable. The maximum consistency between the two methods was from 54% to 96% for field block-2 and shelter block-2, respectively (**Table 3**). In the second scenario, selection is made based on the BLUP values after treatment in both control and treatment conditions, the maximum consistency was 90% for the control and 92% for the treatment conditions, respectively.

**Table 1:** Number of plants in each visual score group in four blocks at two-time points on 15 December 2015 (before treatment) and on 28 July 2016 (after treatment) (visual score 0 indicated dead plant; 1 to 9 indicated lowest to highest vigor scores) from the spaced plant trial.

Date	Visual score	0	1	2	3	4	5	6	7	8	9
15/12/2015	Field block-1	6	3	2	4	9	62	170	248	122	18
	Field block-2	7	2	4	4	10	87	239	231	50	10
	Shelter-1	6	4	4	10	32	140	283	133	30	2
	Shelter-2	4	9	4	8	12	104	290	162	40	11
28/07/2016	Field block-1	20	2	9	14	27	81	157	195	115	24
	Field block-2	23	7	9	10	18	48	109	199	166	55
	Shelter-1	379	13	38	27	42	43	46	27	17	12
	Shelter-2	223	28	43	27	37	54	58	71	69	34

**Table 2:** Top 50 selections based on visual score in four blocks after treatment (number of selection/number of candidate in each group) from the spaced plant trial.

Visual Score	9	8	7
Field block-1	24/24	26/115	–
Field block-2	50/55	–	–
Shelter-1	12/12	17/17	21/27
Shelter-2	34/34	16/69	–

**Table 3:** Top 50 selection based on normalized difference vegetation index (NDVI) from Parrot Sequoia multispectral sensor attached to 3DR Solo Quadcopter in four blocks and their corresponding NDVI range, visual score, and maximum selection consistence between the two methods from the spaced plant trial.

Block	NDVI range	Visual score				Maximum consistency	
		9	8	7	6	No. in common/total	%
Field block-1	0.342–0.399	12	30	7	1	32/50	64
Field block-2	0.320–0.369	27	22	1		27/50	54
Shelter-1	0.243–0.350	12	16	13	9	41/50	82
Shelter-2	0.271–0.374	27	21	2		48/50	96

### Relationship Between NDVI and Biomass From the Row Plot Trial

**Figure 3:**
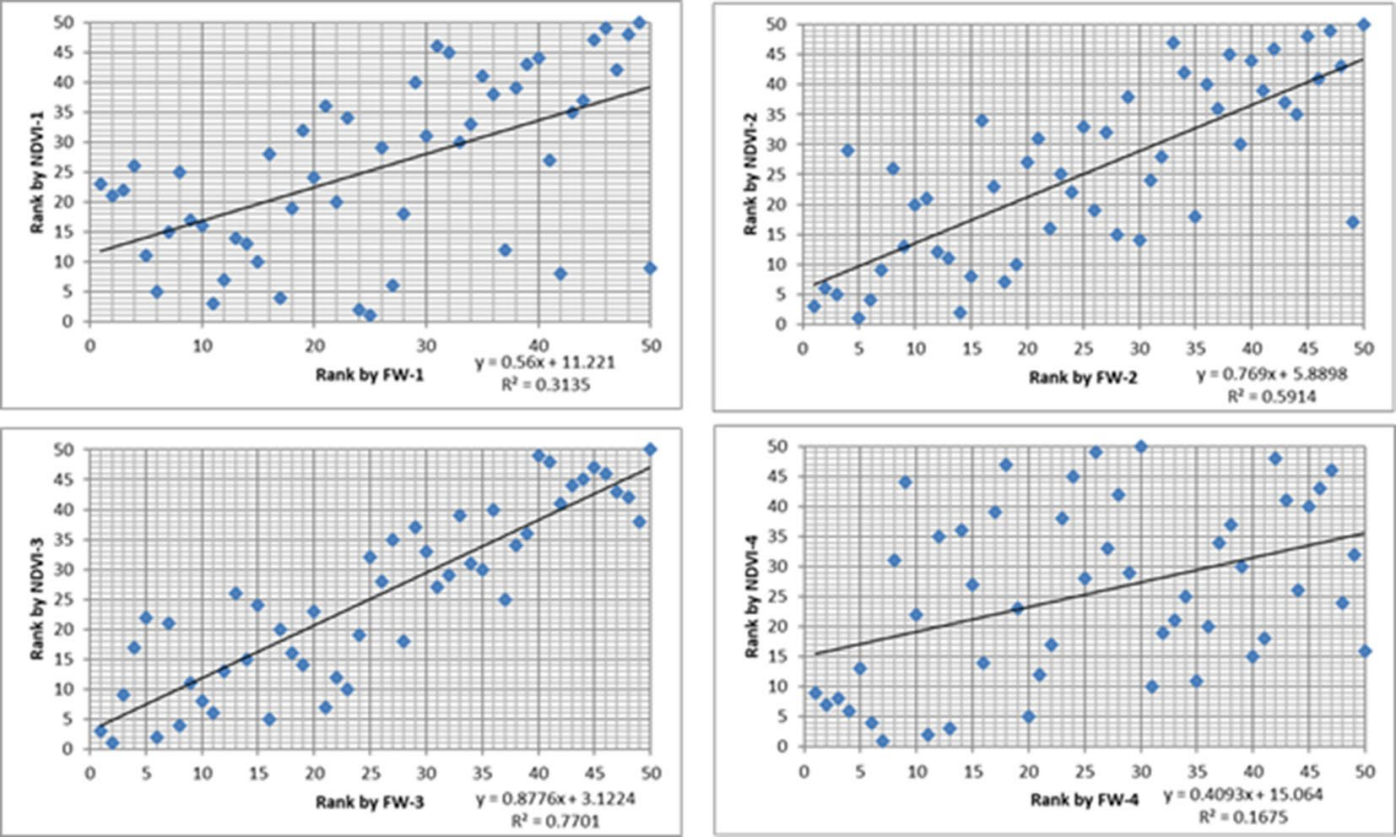
The scatter plots between cultivar ranking based on biomass (x-axis) and ranking based on normalized difference vegetation index (NDVI) (y-axis) from Parrot Sequoia multispectral sensor attached to 3DR Solo Quadcopter in four harvests from the row plot trial.

Significant correlations were observed between biomass yield and aeriel NDVI for the row plot trial (**Table 4**). The correlation coefficients varied from 0.59 to 0.79 in different harvests. The cultivar ranking based on the predicted mean of biomass yield and NDVI was compared (**Figure 3**). Although the correlation of the ranking based on the two measurements were significant for all the 5 harvests (r = 0.45–0.87), the discrepancy of the rank for a particular cultivar was common especially for those middle ranked cultivars. It has to be noted that the different rank order may not necessarily mean any significant difference in cultivar mean yield which will be determined by the least significant difference value. In some cases, the difference was significant. For example, in the first harvest, cultivar C1 was ranked 28^th^ by yield and the yield was significantly less than culitvar C8, which was ranked as 1^st^ (highest yielding cultivar). However, C1 was ranked the 1^st^ by NDVI and not significantly different from C8 which was ranked 6^th^.

**Table 4:** Range of normalized difference vegetation index (NDVI) from NDVI from Parrot Sequoia multispectral sensor attached to 3DR Solo Quadcopter and herbage yield in fresh weight and their correlation coefficent at different cutting date from the perennial ryegrass row plot trial (n = 500).

Harvest	Date	NDVI					FW (kg)					r
		Mean	s.d.	Min	Max	Range	Mean	s.d.	Min	Max	Range	
1	29/11/2016–1/12/2016	0.2382	0.06484	–0.0115	0.3713	0.3598	6.06	1.183	2.4	9.0	6.6	0.27
2	9/05/2017	0.6997	0.05839	0.4967	0.8251	0.3284	2.77	1.051	0.6	6.0	5.4	0.72
3	6–7/07/2017	0.8080	0.03775	0.6389	0.8757	0.2368	7.80	2.325	1.2	14.3	13.1	0.79
4	14/09/2017	0.7555	0.03108	0.6615	0.8388	0.1773	7.54	1.823	3.5	14.9	11.4	0.59

### Relationship Between NDVI and Biomass From Sward Trials

Significant correlations were observed between NDVI and harvested biomass in the cultivar subselection trial from all but one harvest (**Table 5**). The correlation coefficients ranged from 0.12 (May 2015) to 0.91 (October 2015). The correlation coefficients were higher in September and October harvests.

**Table 5:** Correlation coefficent between normalized difference vegetation index (NDVI) from GreenSeeker and herbage yield in fresh weight and dry weight at different cutting date from the cultivar subselection trial (n = 60, NS: not significant at p < 0.05 level. a: NDVI data not available for the first harvest; b: dry weight data not available for these harvests).

Harvest	Cutting Date	NDVI					FW (g)					r	
		Mean	s.d	min	max	Range	Mean	s.d	min	max	Range	NDVI-FW	NDVI-DW
1	3/09/2014						1,601	1,084	79	4,200	4,122	a	a
2	1/10/2014	0.9219	0.01686	0.8718	0.9464	0.0746	2,922	878.1	786	4,373	3,587	0.82	0.78
3	5/11/2014	0.7545	0.05482	0.6113	0.8469	0.2356	2,494	447.8	1,712	3,490	1,778	0.62	0.60
4	27/05/2015	0.7627	0.04844	0.6377	0.8403	0.2026	878	225.4	544	1,626	1,082	0.12^NS^	0.03^NS^
5	30/06/2015	0.8264	0.02717	0.7525	0.8737	0.1212	995	192.8	652	1,457	805	0.57	0.59
6	18/08/2015	0.859	0.03405	0.7682	0.9133	0.1451	1,668	411.5	971	2,601	1,630	0.68	0.67
7	30/09/2015	0.9044	0.02224	0.8647	0.9484	0.0837	3,163	731.1	1,768	5,070	3,302	0.83	0.77
8	28/10/2015	0.6286	0.09014	0.4668	0.8286	0.3618	1,544	528.5	778	2,692	1,914	0.91	0.89
9	14/12/2015	0.2144	0.0238	0.1704	0.3043	0.1339	465	163.3	120	894	774	0.79	b
10	18/04/2016	0.5033	0.06155	0.3608	0.6376	0.2768	317	102.2	63	569	506	0.54	0.50
11	24/05/2016	0.6317	0.08173	0.4293	0.8022	0.3729	403	148.2	161	866	705	0.77	0.74
12	12/07/2016	0.7776	0.06262	0.6214	0.8913	0.2699	685	230.9	247	1,232	985	0.78	0.75
13	6/09/2016	0.6981	0.07257	0.5307	0.8247	0.2940	1,071	343.5	493	1,876	1,383	0.86	0.82
14	19/10/2016	0.7559	0.0508	0.6411	0.8542	0.2131	2,221	684.2	1,131	4,135	3,004	0.82	0.80
15	23/11/2016	0.6933	0.07868	0.5567	0.8377	0.2810	2,623	670.8	1,709	5,023	3,314	0.68	b
16	3/05/2017	0.6617	0.07289	0.5226	0.7913	0.2687	969	408.4	453	2,061	1,608	0.86	0.85

The correlations between NDVI and the biomass were also significant in the perennial ryegrass F_2_ trial for all of the 16 harvests (**Table 6**). The correlation coefficients were from 0.54 (Noveber 2016) to 0.77 (May 2016), which were slightly lower than the cultivar subselection trial.

**Table 6:** Correlation coefficent between normalized difference vegetation index (NDVI) from GreenSeeker and herbage yield in fresh weight and dry weight at different cutting date from the perennial ryegrass F_2_ trial (n = 72, a: data not available due to long harvest peroid; b: dry weight data not available for these harvests).

Harvest	Date	NDVI					FW (g)					r	
		Mean	s.d	Min	Max	Range	Mean	s.d	Min	Max	Range	NDVI-FW	NDVI-DW
1	9/09/2015	0.8455	0.05057	0.6528	0.9498	0.2970	1,070	840.8	147	5,524	5,377	0.63	b
2	30/11/2015–14/12/2015	0.6338	0.06938	0.4936	0.8154	0.3218	1,653	706.9	520	3,140	2,620	a	a, b
3	18/04/2016	0.6903	0.06023	0.5333	0.8093	0.2760	1,003	305.5	431	2,113	1,682	0.67	0.66
4	24/05/2016	0.7892	0.05367	0.6237	0.8713	0.2476	966	320.4	316	1,892	1,576	0.77	0.78
5	15/07/2016	0.8382	0.05393	0.6769	0.9224	0.2455	1,312	499.2	326	2,852	2,527	0.75	0.75
6	6/09/2016	0.7122	0.06445	0.5628	0.8592	0.2964	1,452	639	481	3,988	3,507	0.74	0.71
7	13/10/2016	0.7741	0.0454	0.6576	0.8841	0.2265	1,816	622.9	99	3,935	3,836	0.67	0.62
8	15/11/2016	0.7688	0.04287	0.6456	0.8669	0.2213	2,180	484.9	1,181	3,498	2,317	0.54	0.46
9	19/12/2016	0.5889	0.09472	0.3897	0.8317	0.4420	1,437	586.4	570	3,745	3,175	0.64	b
10	1/02/2017	0.467	0.06305	0.3622	0.7113	0.3491	648	300.7	294	2,219	1,925	0.60	b
11	26/04/2017	0.6591	0.04814	0.5292	0.7988	0.2696	934	262.7	430	1,674	1,245	0.56	0.49
12	21/06/2017	0.8875	0.0321	0.8136	0.9578	0.1442	1,819	772	570	3,899	3,329	0.75	0.72
13	20/09/2017	0.6212	0.05318	0.5158	0.7817	0.2659	1,649	607.4	790	3,764	2,974	0.68	0.57
14	14/11/2017	0.7668	0.05249	0.6654	0.8716	0.2062	3,030	936.8	1,416	6,062	4,646	0.61	0.53
15	9/01/2018	0.5364	0.08475	0.399	0.8411	0.4421	1,220	591.8	440	3,211	2,772	0.51	0.46
16	24/05/2018	0.427	0.0527	0.304	0.57	0.2660	395.2	205.7	67.4	997.1	930	0.57	0.53

**Figure 4:**
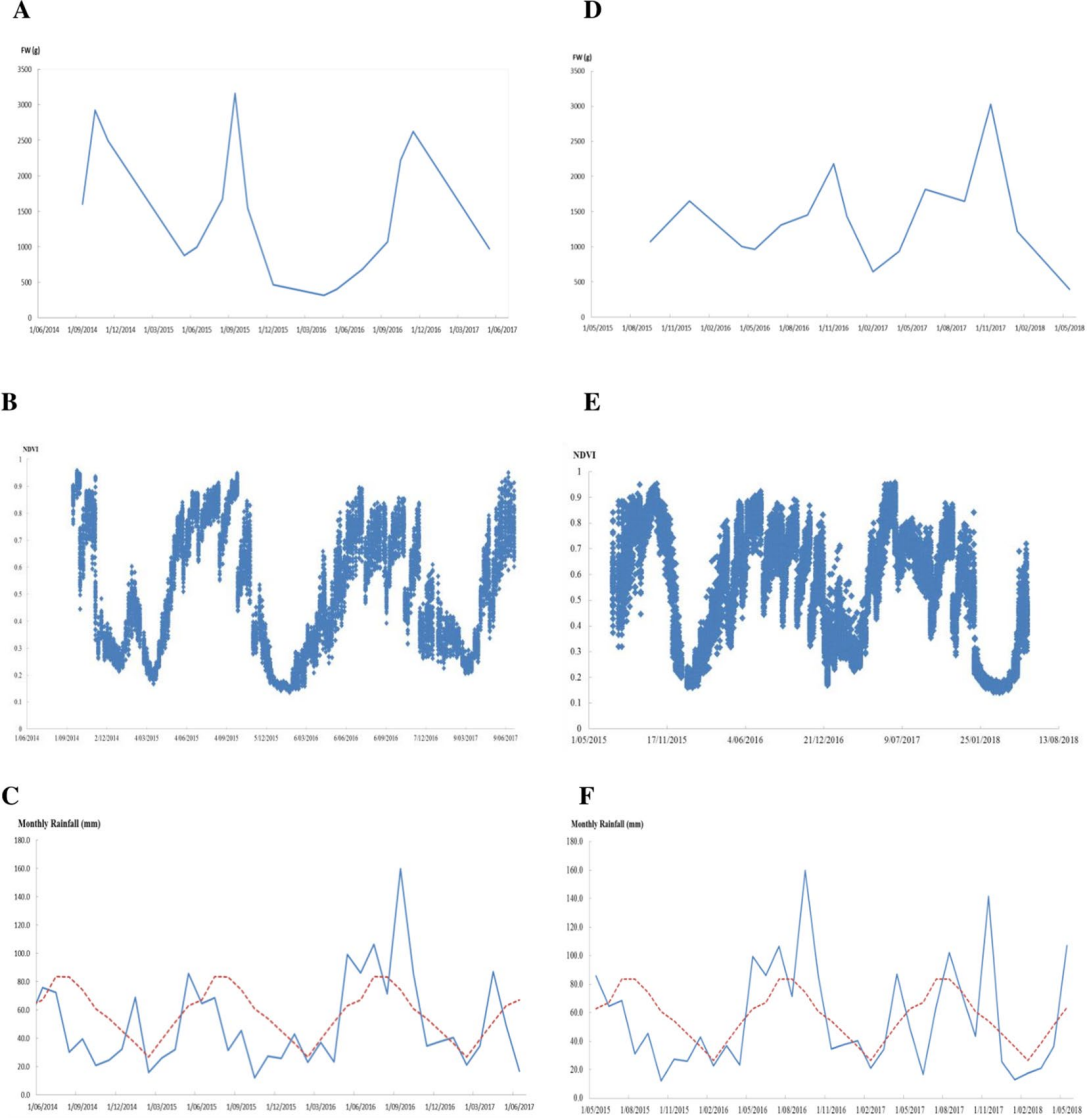
Biomass and normalized difference vegetation index (NDVI) from GreenSeeker change overtime and the monthly rainfall during the experimental periods in the cultivar sub-selection trial **(A**–**C)** and perennial ryegrass F_2_ trial **(D**–**F)**, respectively (red dotted lines in **(C** and **F)** indicate long-term average rainfall).

Monitoring NDVI weekly during the 2.5-year experimental peroid for perennial ryegrass sward trials showed marked seasonal changes of the NDVI values (**Figures 4B, E**). NDVI was at the lowest point during summer and gradually increased and reached its peak in winter and early spring, then gradually decreased again into summer. The trend of this seasonal change largely followed the pattern of seasonal yield in this environment (**Figures 4A, D**) and the monthly rainfall pattern of the trial site (**Figures 4C, F**). The unusually high rainfall that occurred during the summer in January 2015 was associated with a clear transient NDVI peak while there was no such peak in the summer of 2016 and 2017 when there was a normal rainfall pattern with little summer rain. In the summer time, ryegrass plants are generally dormant, NDVI values were low, and differences between cultivars were small. In the winter and spring, NDVI values were higher and difference between cultivars were also larger (**Figures 4B, E**). There were sharp declines of NDVI in the spring in 2014 and 2015 in both trials due to lower than long-term average rainfall in contrast to the much slower decline of NDVI in the spring of 2016 where above long-term average rainfall occured.

## Discussion

To accommodate the need to collect a large amount of relatively accurate yield data rapidly and cost effectively, forage breeders select breeding parents based on visual vigor score of genotypes. For mass selection, nursery trials with up to 10,000 plants are not uncommon (Hayes et al., 2013). Scoring tens of thousands of plants in a field with a pen and a notepad is a tiring task and may take a few days. With sensors and UAS, it takes less than 20 minutes to fly over the site to capture the data to rank plants based on vegetative indices. In addition, in perennial grass breeding, programs scoring needs to happen at multiple times throughout multiple years to capture seasonal yield changes. The time saving of the application of sensor-based screening technology is enormous. The other advantage is that the data is stored electronically and can be checked and retrieved anytime afterwards. Most importantly, the multispectral image is more informative than the simple visual score and rank and it gives precise, continuous value of indices hence much higher resolution. The rapid adoption and increasing affordability of UAS and sensors provides potential for routine application of this technology in breeding. The major concern currently is the analysis and data extraction from large volumes of data. With the development of some open sourced and licensed computer programs such as those used in this study, image analysis is becoming more streamlined.

The significant correlation between NDVI and visual score forms the basis for NDVI to replace visual score. It was noted that there were overlaps of NDVI range across different ranking groups. For example, visual score as “0,” which indicated a dead plant, with a range of NDVI values 0–0.1 in 2015 and 0–0.2 in 2016. This was partially due to leaves of neighboring plant that fill into the polygon delineated the plant. So, the accuracy of NDVI for each plant will depend on how well the segmentation of the image matches the actual plant. In this spaced plant trial, the distance between neighboring plants within a column was 40 cm which was less than the common distance in breeders’ nursery trial which is 50 or 60 cm. The greater the distance, the clearer of the separation between the neighboring plants and more accurate of the NDVI value. In this experiment, we extracted data automatically without manual correction, which would be useful if higher accuracy was required. Selection of genotypes based on NDVI and selection based on visual score was largely in agreement. The bigger the difference is between the genotypes the higher the confidence is for visual score and the higher consistency between the two methods as seen in the stressed condition compare to the control condition. The high-throughput nature of this technology may allow more replication within sites which will reduce environmental variance and improve selection efficiency. The NDVI obtained from multispectral sensors and UAS can replace visual score to assist selection.

Highly significant correlations were observed between NDVI and the biomass yield at row plot level. A similar correlation (r = 0.79) between biomass yield and NDVI was observed in wheat row trial (Tucker et al., 1981). The ranking of the 50 cultivars was correlated between based on biomass and based on NDVI although the rank discrepancy occurred commonly for a specific cultivar (**Figure 4**). However, it must be noted that even among cultivars with different ranks, the difference in yield may or may not be significant. At the current level of correlation (r = 0.59 to 0.79), some cases of true difference existed in ranks of a cultivar based on NDVI and the yield. Therefore, cultivar rank by aerial NDVI was not always in complete agreement with yield rank. The missing accuracy may be due to the saturation of NDVI value at high density vegetation (Gu et al., 2013). Beyond the saturation point, major component of yield may be explained by combination with other terms such as canopy height and structure. For perennial pasture, the saturation NDVI value may provide a threshold point for grazing rotation and this requires further study. Another reason may be the intensity of leaf green color difference between cultivars, which ranges from very light green to very dark green in perennial ryegrass (UPOV), may cause differences in reflectance. This may make the cross-cultivar comparison less accurate. In this circumstance, the NDVI model could be adjusted according to different cultivars to achieve better ranking agreement with biomass.

Significant correlations were observed between the ground-based NDVI and biomass for all 32 harvests except one from the two sward trials. The lack of correlation between NDVI and herbage yield in harvest 4 of the cultivar subselection trial (**Table 5**) may be due to the skewed distribution of yield (skewness 1.16). Under the climatic conditions of the trial site, the seasonal change of perennial ryegrass pasture production is remarkable. The NDVI change over time reflected the seasonal yield trend and may be explained since NDVI has been considered as an indicator of “greenness” (chlorophyll content) and positively correlated with photosynthetic rate in plants where canopy development and photosynthetic activity were in synchrony (Gamon et al., 1995). The NDVI fluctuation was also associated with the rainfall events and was more prominent when unusual rainfall occurs. So, plant response to moisture was reflected by the NDVI change. The NDVI may serve as a good early indicator of plants responses to the environment, hence to explore genotype-environment interaction during cutting/grazing intervals and to track regrowth after cutting/grazing to explore the dynamics and persistence of perennial pastures. Multispectral sensors and UAS also allow tracking productivity over time for perennial pasture, in particular, the regrowth after grazing or clipping and to explore seasonal changes and responses to environmental factors such as precipitation. We found that the correlation coefficients changed over harvests/seasons. In summer time, the correlation was generally lower than in spring, autumn, and winter. Similar trends have been reported by Schino et al. (2003), who indicated that in summer when the ratio of dry/green biomass increases, NDVI estimate becomes less accurate.

In this paper, we validated the most common vegetation index NDVI in the three main types of early-generation grass breeding trials for correlation with the visual score and biomass. Aerial NDVI was significantly correlated with visual score and has clear advantages and may replace visual score in forage grass breeding programs. NDVI was significantly correlated with the biomass yield in row plot trial and simulated sward trials. Cultivar rank by NDVI was correlated with the rank by yield. Future research would be to explore ways to improve the accuracy for biomass estimation. The modeling of absolute biomass prediction is not the focus of this paper, but would be important in predicting and monitoring animal consumption and production and for estimation of yield in larger sward trials. To accurately model biomass prediction, other terms including height, volume, and density may be measured simultaneously in combination with other sensors such as sonar and LiDAR (Deery et al., 2014). Remote sensing for traits related to forage quality have been investigated and with mixed results for different parameters (Starks et al., 2004; Guo et al., 2010). Further research would be preferable into nondestructive forage quality evaluation procedures for ready application. NDVI provides an indication to study the plant response to environmental factors over time without destructive intervention. The sensors together with ground- and aerial-based platform technologies may be extended to pastoral farmland management in collection of data on the germination rate, early vigor, speed of establishment, and spatial variations to assist with decision making and to contribute to digital and precision agriculture.

## Data Availability Statement

All datasets generated for this study are included in the article/supplementary material.

## Author Contributions

GS, NC, KS, and PB conceived the research. JW designed the field trials, analyzed data, and drafted the paper. PB and AP conducted the flight and analyzed images. LP performed the image segmentation for the spaced plant trial. FS performed the image segmentation for the row plot trial.

## Conflict of Interest

The authors declare that the research was conducted in the absence of any commercial or financial relationships that could be construed as a potential conflict of interest.
